# Overcoming the challenge of COVID‐19: A grounded theory approach to rural nurses' experiences

**DOI:** 10.1002/jgf2.410

**Published:** 2020-11-29

**Authors:** Ryuichi Ohta, Yaeko Matsuzaki, Satomi Itamochi

**Affiliations:** ^1^ Community Care Unnan City Hospital Unnan Japan; ^2^ Department of Nursing Unnan City Hospital Unnan Japan

**Keywords:** COVID‐19 ward, identity crisis, Japan, nurses, rural hospital

## Abstract

**Introduction:**

This study inquires into nurses' changing perceptions with regard to the efforts in preparation for working in a COVID‐19 ward in the rural Japanese context.

**Methods:**

Data were collected using ethnographic methods and semi‐structured interviews among 16 nurses working in the COVID‐19 ward of a rural community hospital in Japan. A grounded theory approach was used for the qualitative analysis.

**Results:**

In total, 70 hours' observation and participation were completed, and 27 pages of field notes were taken and used for the analysis. In addition, 32 interviews were conducted with 16 participants. Four themes emerged from the data: pre–COVID‐work perceptions, overcoming fear, shadow cast by working in the COVID‐19 ward, and an integrated approach to the fear of COVID‐19. The nurses initially felt unpredictable fear. However, the establishment of standard approaches and practices for COVID‐19 gave them confidence in their safety and helped them regain sympathy for patients. Nevertheless, working on COVID‐19 cases negatively affected their activities outside of the ward, and some of them developed an identity crisis as they feared for the future.

**Conclusion:**

Better teamwork, comprehensive understanding of COVID‐19, and continuous provision of proper knowledge in rural hospitals should be driven by appropriate understanding and sympathy for nurses and patients in COVID‐19 wards. The results of this study can be applied to mitigate nurses' fear, improve teamwork, and ensure understanding of COVID‐19 by all medical staff in rural hospitals.

## Graphical abstract

This study clarified rural nurses' experiences in the COVID‐19 ward. For overcoming the challenges of COVID‐19, comprehensive approaches of community hospitals are needed.

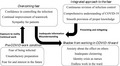

## 1 INTRODUCTION

Different countries have taken different approaches to addressing the rise in infection rates from the novel coronavirus disease (COVID‐19) pandemic.^1^ Many countries have experienced large numbers of COVID‐19 patients and causalities.^2^, ^3^ Medical institutions and systems have been overwhelmed, and many medical professionals have been infected. The stress of fighting COVID‐19 has also caused physical and mental health problems among medical staff.^4^ In previous pandemics, healthcare professionals have suffered from stress caused both indirectly by pandemic‐related social issues^5^, ^6^, ^7^ and directly by medical issues such as large numbers of causalities in intensive care units (ICUs).^8^, ^9^ Pandemic experience can facilitate healthcare workers' motivation and effectiveness in clinical situations.^10^, ^11^

The COVID‐19 pandemic has mobilized medical institutions globally, including healthcare professionals not used to controlling infection, in both urban areas and rural areas where pandemic experience may be less, leading to greater stress dealing with COVID‐19.^12^ Medical staff in rural community hospitals have had to quickly learn to effectively manage patients with COVID‐19.^13^ Non–evidence‐based information from social media and news sources has also induced stress and prejudice among medical staff dealing with COVID‐19.^14^, ^15^ Staff working in COVID‐19 wards can find it psychologically difficult to interact with other medical staff for fear of infecting them, even if they are well prepared for COVID‐19 patients.^16^

In rural Japanese areas, lack of medical resources means COVID‐19 care can impinge on healthcare work conditions. Healthcare workers' physical and mental condition should be attended to carefully as part of effective management response. However, there is little research on rural nurses' perceptions regarding COVID‐19, although preparations for the next wave of COVID‐19 or subsequent pandemics could be facilitated by research that helps us understand the distinctive situation of rural areas vis‐à‐vis COVID‐19. This research examined nurses' changing perceptions of preparing for COVID‐19 and working in COVID‐19 wards.

## 2 METHODS

### 2.1 Setting

Unnan is a small, remote, rural city in Japan. In 2020, the total population was 37,638 (18,145 males and 19,492 females), of whom 39% were aged over 65; this is expected to reach 50% by 2025. The city has 16 clinics, 12 homecare stations, 3 visiting nurse stations, and a single public hospital. At the time of the study, Unnan City Hospital had 281 care beds, of which 160 were dedicated to acute care, 43 to comprehensive care, 30 to rehabilitation, and 48 to chronic care. The nurse‐to‐patient ratio was 1:10 in acute care, 1:13 in comprehensive care, 1:15 in rehabilitation, and 1:25 in chronic care. The hospital had 27 physicians, 197 nurses, 7 pharmacists, 15 clinical technicians, 37 therapists, 4 nutritionists, and 34 clerks. Most hospital health workers were from the city.

### 2.2 Participants

Participants were 16 nurses who worked in a COVID‐19 ward. They had previously worked in general departments in the hospital or in wards specializing in infections. After one ward was converted to a COVID‐19 ward, they were voluntarily assigned to it after being provided with general information about COVID‐19 and the necessity of wearing N95 masks and PPE (personal protective equipment) by a nurse specializing in infectious diseases. Next, they prepared the ward for COVID‐19 by dividing it into safe, semi‐infectious, and infectious zones. After they had practiced managing COVID‐19 by simulation, the ward started accepting patients. Working in the ward, the nurses discussed and revised their care management practices collaboratively and discussed them with infectious disease nurses.

COVID‐19–dedicated nurses were bunked in a hospital dormitory and provided free meals by the hospital. Their movement was not restricted, and they used the same rooms as other medical staff to change clothes for work.

### 2.3 Ethical considerations

Before providing written consent, participants were informed that the data would only be used for research purposes. They were also informed about the research aims, data disclosure procedures, and steps taken to protect personal information. This study was approved by the Unnan City Hospital Clinical Ethics Committee (approval number: 20200005).

### 2.4 Measurement

Ethnography and semi‐structured interviews with participants were conducted by the first author, a specialist in family medicine, medical education, and public health, charged with treatment of COVID‐19 patients. This author participated in the nurses' activities, such as cleaning the ward and delivering food to patients, observing nurses' work and approaches to patients with COVID‐19 for 3 days, participating in discussions of care management, and taking field notes. Next, the researcher interviewed the participants regarding their work in the COVID‐19 ward. Three main questions were asked: *How did you feel about working in this ward before being assigned here? How do you feel about your present working conditions?* And *do you feel that there have been any difficulties or that any changes should be made regarding working here?* Each interview lasted about 30 minutes and was recorded, transcribed verbatim, and reviewed and confirmed by the interviewee. Participants also wrote down the challenges they faced and their suggestions for better processes and shared them with the researcher.

### 2.5 Data analysis

Grounded theory was used to identify changes in nurses' perceptions of preparation for COVID‐19 and working in the COVID‐19 ward. The first and second authors carefully read the field notes, transcriptions, and participants' difficulties and suggestions. The first author then coded the content and developed codebooks based on repeated reading (initial coding).^17^ The study used process and concept coding.^18^ The second author also coded the materials and discussed the coding and codebooks with the first author; on this basis, the authors inducted, merged, deleted, and refined concepts and themes by oscillating between research materials and initial coding in the subsequent axial coding.^17^ Discussion of data and coding continued until mutual agreement was reached and no new codes or concepts appeared. For member checking, the analysis was provided to all participants, whose feedback was included in the final revision of the themes and concepts. Eventually, no new themes emerged during member checking, indicating saturation. Finally, the theory was discussed and agreed upon by all the authors.

## 3 RESULTS

**FIGURE 1:**
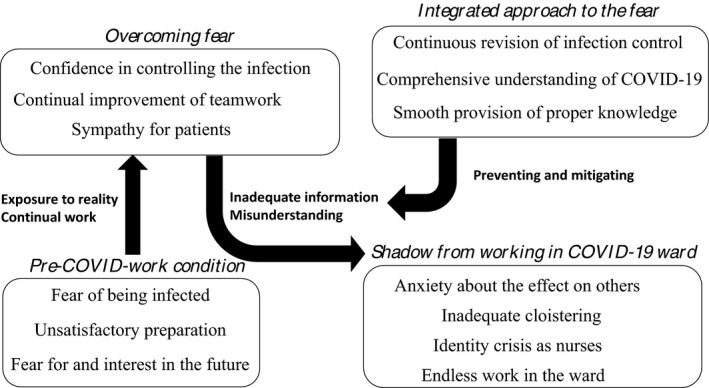
Conceptual model of the process of changes in nurses' perceptions

Overall, 70 hours of observation and participation was performed, 27 pages of field notes were written and analyzed, and 32 interviews were conducted with 16 participants (each interviewed twice). Through grounded theory, four themes emerged: *pre–COVID‐work perceptions*, *overcoming fear*, *shadow cast by working in the COVID‐19 ward*, and *integrated approach to the fear of COVID‐19*. Figure 1 presents a conceptual model of this study's results.

### 3.1 Pre–COVID‐work perceptions and fear

#### 3.1.1 Fear of being infected

Prior to working in the COVID‐19 ward, the participants experienced fear of COVID‐19 as a result of vague evidence about this unprecedented health event and felt anxious owing to the possibility of infection in the ward. As they were exposed to varied information through television, the Internet, and their families and friends, their anxiety and concern about working in the ward grew.

I was exposed to too much information regarding this infection. The contents were hectic and fearful for me. I was motivated to work at this ward, but the information from friends and family could have also prevented me from going to this ward because of their fear of COVID‐19. These complicated situations confused me, leading to more fear of working here. (Participant 4)

#### 3.1.2 Unsatisfactory preparation

As COVID‐19 spread quickly in Japan, local governments acted rapidly, setting up special beds and wards. Participants felt preparations had been so quick that they had not prepared themselves adequately for COVID‐19 before being expected to treat it. Stress interfered with their efficient absorption of required infection control knowledge.

#### 3.1.3 Fear for and interest in the future

Participants experienced anxiety about the future and felt it hard to return to their previous jobs owing to lost experience with regular nursing care. Some participants wished to continue working in the COVID‐19 ward; although they felt fear, they were motivated to help fight this new disease.

We thought that we were not prepared to deal with the patients of COVID‐19 because of the lack of time for preparation. As the speed of the Japanese and local governments with the preparation for COVID‐19 was so high, we could not master the processes of dealing with patients of COVID‐19 and felt fear about our future. (Participant 2)

Although the pandemic was a hectic situation, I was interested in this unprecedented situation. Of course, my family disagreed with my idea. Nevertheless, I thought that I had never experienced this working situation, so I decided to work here. (Participant 6)

### 3.2 Overcoming fear

#### 3.2.1 Confidence in controlling the infection

After starting work in the ward, participants realized that most patients with COVID‐19 did not have severe symptoms and did not need frequent intervention. Thus, firsthand experience made them confident that they would not be infected too easily. In addition, their anxiety regarding unsatisfactory preparations was dispelled by the frequent experience of wearing PPE and N95 masks and moving around the ward within the zoning setup.

#### 3.2.2 Continuous revision of infection control

Participants discussed care methods for COVID‐19 patients, the difficulty of their work, and the danger of infection. In the process, they came to better understand each other, which improved their teamwork and collective understanding and reduced their stress and anxiety about infection.

I noticed that this ward was very clean, and the risk of infection was low. [Description of care processes.] Most of us have been educated and worked together in other words, so we know each other and can discuss smoothly. Furthermore, we continuously discuss care management in this ward among ourselves. Through the process, we can make revisions to the management procedures and understand others' perception of the present conditions. (Participant 14)

#### 3.2.3 Sympathy for patients

As participants worked closely with patients, their usual sympathy for patients returned. Although all patients had mild symptoms, they could not go out because of the risk of transmission. Nurses hoped the patients would be discharged soon after testing negative for COVID‐19.

I was exhausted with the fear of COVID‐19, so I was not able to develop empathetic feelings towards the patients to protect my safety. Talking with the patients, I began to feel sympathy for them soon after feeling secure. I hope that they can be discharged from here by getting a negative result for COVID‐19 soon. (Participant 7)

### 3.3 Shadow cast by working in the COVID‐19 ward

#### 3.3.1 Anxiety about effect on others

After their fear of self‐infection declined, nurses became anxious about the potential effects of working in the COVID‐19 ward on people they came into contact with. They felt distant from staff in other wards and suspected that their colleagues might avoid approaching them and consider them infected even if they had no symptoms. Further, some participants developed anxiety about their family's condition and segregation from schools and workplaces. Social media and TV increased their anxiety, and they realized their working conditions were not adequately understood by other staff.

Now, I do not feel anxiety about getting infected myself. Although this can be difficult to understand, I am anxious about the possibility of the transmission of infection to others even if I know I am not infected. Furthermore, I sometimes felt medical staff working at other wards avoided me. It may be the effect of what the social media and TV say about COVID‐19. (Participant 10)

I fear that my family can be discriminated against in the community because of my working conditions. Although medical staff understand the conditions of controlling infection, the administration staff may not understand the evidence or have proper knowledge of COVID‐19. I am always pressing my family to tell me whether they are being ostracized by others or feel that they are segregated from others. (Participant 12)

#### 3.3.2 Inadequate cloistering

Participants tended to cloister themselves and avoid approaching colleagues in other wards because of general perceptions of COVID‐19 and workers in the COVID‐19 ward. Some of them had experienced public rejection when trying to use services such as shopping or hair salons. This led to depressive feelings and anxiety in their private life.

I do not have any motivation to go out of my apartment because I do not meet colleagues in public places. Meeting me can cause them to have negative emotion. I had experienced rejection from the hair salon when I confessed my working situation. Eventually, thanks to the chief of the hair salon, I got service. However, at the present moment, I do not go anywhere, even on a day off. (Participant 5)

#### 3.3.3 Identity crisis as nurses

As participants built good relationships with patients, they tried to work with patients more closely—a core part of nursing work and identity. However, risk of infection meant they also needed to maintain distance from patients, which led to identity crisis as nurses. They realized that touching and listening to patients, aside from building better relationships, was vital to their professional identity, which they hoped to regain.

We can usually make good relationships with patients by touching and listening to them at short distances. However, now we do not act like that. Although we understand the present situation, we are unintentionally approaching patients. We have to restrain our identity as nurses. (Participant 2)

### 3.4 Integrated approach to fear

#### 3.4.1 Continuous improvement of teamwork

To moderate participants' stress and anxiety, collaboration in the ward was essential. While working, participants shared ideas and experiences and encouraged each other. Mutual understanding mitigated their stress and anxiety, stimulated teamwork, and potentially prevented them from experiencing depression.

#### 3.4.2 Comprehensive understanding of COVID‐19

Negative feelings about the nurses among other medical staff were elicited by the latter's poor understanding of COVID‐19. The COVID‐19 ward was established so quickly that most medical staff were not appropriately informed about COVID‐19 and the required ward structure. Participants indicated that comprehensive understanding of COVID‐19 among stakeholders might mitigate negative feelings toward the ward.

We established effective teamwork in this ward. Here, we can share various ideas and motivate each other to work. This teamwork should be driven to maintain a good mental condition of the nurses. Besides, a larger number of hospital staff should be aware of the condition of this ward. Their misunderstanding can cause misconduct towards this ward's nurses. Although nurses of this ward cannot work as hard as they used to previously, we hope to be treated more naturally, because we do not experience severe working conditions. (Participant 14)

#### 3.4.3 Smooth provision of proper knowledge

Timely, correct, locally specific COVID‐19 information is vital to help nurses manage fear of COVID‐19. Government‐provided COVID‐19 information changes daily, and nurses and other medical professionals should be kept informed.

## 4 DISCUSSION

This study clarified changes in perceptions among nurses working in a COVID‐19 ward of a rural hospital in Japan and explained their views on potential resolutions. Four themes emerged: pre–COVID‐work perceptions, overcoming fear, shadow cast working in the COVID‐19 ward, and integrated approach to fear of COVID‐19. Nurses working in COVID‐19 wards had previously felt unpredictable fear regarding COVID‐19, inducing various negative perceptions; after working in the COVID‐19 ward, they established and improved methods for approaching COVID‐19, acquired confidence in their safety, and regained sympathy for patients. However, working in the COVID‐19 ward also negatively affected their activities outside of the ward, inducing identity crisis and anxiety about the future.

COVID‐19 fear was caused by poor knowledge of the disease and insufficient time to prepare special COVID‐19 wards. As the participants stated, overwhelming information regarding the pandemic impinged on their preparation of work in the COVID‐19 ward. Pandemic situations expand the volume of health information, which can cause confusion and misunderstanding.^19^, ^20^ The situation might be exacerbated by a large proportion of older people in rural settings, which may cause high mortality rate if infected.^1^ As this study's participants stated, in their situation, where rural people could not acquire proper information, caring for patients with COVID‐19 exacerbated fear of infection.^21^ At the same time, pandemics are shown to stimulate rural nurses to work^8^, ^22^; professionalism can drive motivation to protect their hospital and decide to work in the infection ward—especially here, as most of the nurses were indigenous to the city.

Rural nurses in COVID‐19 wards adjusted their working conditions through effective collaboration. As the participants stated, by applying required guidelines and subscribing to the advice of infection control nurses and epidemiologists, they understood the situation better and reduced their fear of the unexplainable,^23^ repeating, and routinizing actions for infection control.^24^ In addition, revision of infection control methods based on nurses' experiences and guidelines enabled development of infection control processes more suitable for the ward's conditions.^25^ As participants also stated, their hospital is relatively small, and most nurses have worked together and know each other. This situation can facilitate their preparation for working and revision of working conditions. Furthermore, results showed that by controlling their fear, nurses could consider patients' anxiety and difficulties around conducting self‐care even with little face‐to‐face communication among medical staff. These changes in nurses' perceptions of patients with COVID‐19 enabled them to regain their usual sympathetic outlook.

Even after adapting to COVID‐19 work, participants felt identity crisis as nurses and anxiety living in the community. The gap between the perceptions and the capability to work in a COVID‐19 ward might impair nurses' competence, leading to identity crisis around continuing work or education even after the pandemic has receded.^26^ ^,^ ^27^ Previous research suggests that emergency situations can elicit identity crises among nurses in the Japanese context.^28^ In this study, nurses often cared for older people with multimorbidity, which needed close care and physical touch and felt identity crisis owing to a sense of inadequacy to conduct patient care. However, continuous endeavor to improve conditions and processes of treatment can improve nurses' perceptions and redress this impairment.^28^, ^29^

Being in the COVID‐19 ward can also induce anxiety that one will be viewed with fear by others, causing one to cloister oneself. In this research, fear of COVID‐19 infection was accompanied by emotional stress based on the imaginary perceptions of others and the risk of discrimination; these findings have also been corroborated by other studies in COVID‐19^30^ and non–COVID‐19 contexts.^31^ As the participants stated, the distance the nurses felt from others in and outside of the hospital made them cloister themselves, feel depressed, and fear for the future, even after finishing work in the COVID‐19 ward. This situation is probably exacerbated in rural contexts, where strong social norms make citizens, including medical professionals, more conscious of others' perspectives and reluctant to venture out when this action may have negative consequences. Also, rural nurses' privacy may not be properly protected, and they might fear the rejection from their communities.^22^ Thus, special management of mental health for nurses on the frontlines may be needed in pandemic conditions.^32^, ^33^

Continuous efforts to improve teamwork are vital to mitigate fear and stress among COVID‐19 nurses. As this study's participants stated, a friendly and reliable relationship among working staff results in effective, efficient work, and also decreases nurses' stress and fear by giving them chances to express themselves freely and share their feelings regularly with colleagues, which can also help build productive, sustainable working relationships.^34^ Such solutions should be provided to staff not only in COVID‐19 wards but also throughout the hospital. As for information provision, most medical staff are not infection specialists and are unable to choose appropriate information resources from among the varied information available on COVID‐19.^35^ The use of different information resources by professionals in the same hospital can confuse management; conversely, appropriate information management by infection teams can benefit staff's knowledge,^36^ mitigate their fear, and motivate them to work safely.^37^

One of this study's limitations is that the interviewer was a physician in the same hospital as the nurses. This might have led nurses to be less critical of physicians in their responses. Also, the interviewer might not have fully understood the nurses' tasks, which could have led to irrelevant interview questions being asked.^37^ To overcome this limitation, the interviewer observed the participants and participated in their work in the COVID‐19 ward for 3 days to understand their daily tasks. Another limitation relates to differences in the COVID‐19 pandemic situation worldwide; the impact in Japan is mild as compared to other countries. The results of this study can be applied to mitigate nurses' fear, improve teamwork, foster understanding of COVID‐19 by all hospital staff, and ensure continuous provision of proper knowledge. These actions can drive comprehensive approaches to COVID‐19 in rural hospitals and generate appropriate understanding and sympathy for nurses and patients in COVID‐19 wards.

## 5 CONCLUSION

This study has clarified changes in the perceptions of nurses in the COVID‐19 ward of a rural hospital in Japan and explained their views on potential responses. Grounded theory revealed four themes: pre–COVID‐work perceptions, overcoming fear, shadow cast working in the COVID‐19 ward, and integrated approach to fear of COVID‐19. Nurses working in COVID‐19 wards previously felt unpredictable fear and negative perceptions regarding COVID‐19; after working in the ward, they established and improved methods for approaching COVID‐19, acquired confidence in their safety, and regained sympathy for patients. However, working in the COVID‐19 ward also negatively affected their activities outside of the ward, inducing identity crisis and anxiety about the future.

## CONFLICT OF INTEREST

The authors have stated explicitly that there are no conflicts of interest in connection with this article.
